# Differentiation of Human Embryonic Stem Cells to Sympathetic Neurons: A Potential Model for Understanding Neuroblastoma Pathogenesis

**DOI:** 10.1155/2018/4391641

**Published:** 2018-11-01

**Authors:** Jane Carr-Wilkinson, Nilendran Prathalingam, Deepali Pal, Mohammad Moad, Natalie Lee, Aishwarya Sundaresh, Helen Forgham, Peter James, Mary Herbert, Majlinda Lako, Deborah A. Tweddle

**Affiliations:** ^1^Wolfson Childhood Cancer Research Centre, Northern Institute for Cancer Research, Newcastle University, UK; ^2^North East Stem Cell Institute, Newcastle University, UK; ^3^Faculty of Health Sciences and Wellbeing, University of Sunderland, UK; ^4^Institute of Genetic Medicine, Newcastle University, UK; ^5^Northern Institute for Cancer Research, Paul O-Gorman Building, Newcastle University, UK; ^6^Institute of Health & Society, Newcastle University, UK; ^7^Newcastle Fertility Centre, Newcastle University, UK; ^8^Great North Children's Hospital, Newcastle upon Tyne Hospitals NHS Trust, UK

## Abstract

**Background and Aims:**

Previous studies modelling human neural crest differentiation from stem cells have resulted in a low yield of sympathetic neurons. Our aim was to optimise a method for the differentiation of human embryonic stem cells (hESCs) to sympathetic neuron-like cells (SN) to model normal human SNS development.

**Results:**

Using stromal-derived inducing activity (SDIA) of PA6 cells plus BMP4 and B27 supplements, the H9 hESC line was differentiated to neural crest stem-like cells and SN-like cells. After 7 days of PA6 cell coculture, mRNA expression of *SNAIL* and *SOX-9* neural crest specifier genes and the neural marker *peripherin* (*PRPH*) increased. Expression of the pluripotency marker *OCT 4* decreased, whereas *TP53* and *LIN28B* expression remained high at levels similar to SHSY5Y and IMR32 neuroblastoma cell lines. A 5-fold increase in the expression of the catecholaminergic marker *tyrosine hydroxylase (TH*) and the noradrenergic marker *dopamine betahydroxylase (DBH*) was observed by day 7 of differentiation. Fluorescence-activated cell sorting for the neural crest marker p75, enriched for cells expressing *p75*, *DBH*, *TH*, and *PRPH*, was more specific than p75 neural crest stem cell (NCSC) microbeads. On day 28 post p75 sorting, dual immunofluorescence identified sympathetic neurons by PRPH and TH copositivity cells in 20% of the cell population. Noradrenergic sympathetic neurons, identified by copositivity for both PHOX2B and DBH, were present in 9.4% ± 5.5% of cells.

**Conclusions:**

We have optimised a method for noradrenergic SNS development using the H9 hESC line to improve our understanding of normal human SNS development and, in a future work, the pathogenesis of neuroblastoma.

## 1. Introduction

The neural crest is a transient embryonic cell population which undergoes extensive migration and differentiation to give rise to a diverse range of cell populations in the embryo, ranging from the peripheral nervous system (sensory, enteric, and autonomic (sympathetic and parasympathetic)) to the craniofacial skeleton and pigment cells (reviewed by [1]). Neural crest cells are multipotent stem cells which can self-renew and in humans undergo extensive migration around the third to fourth weeks of gestation [2].

**Figure 1:**
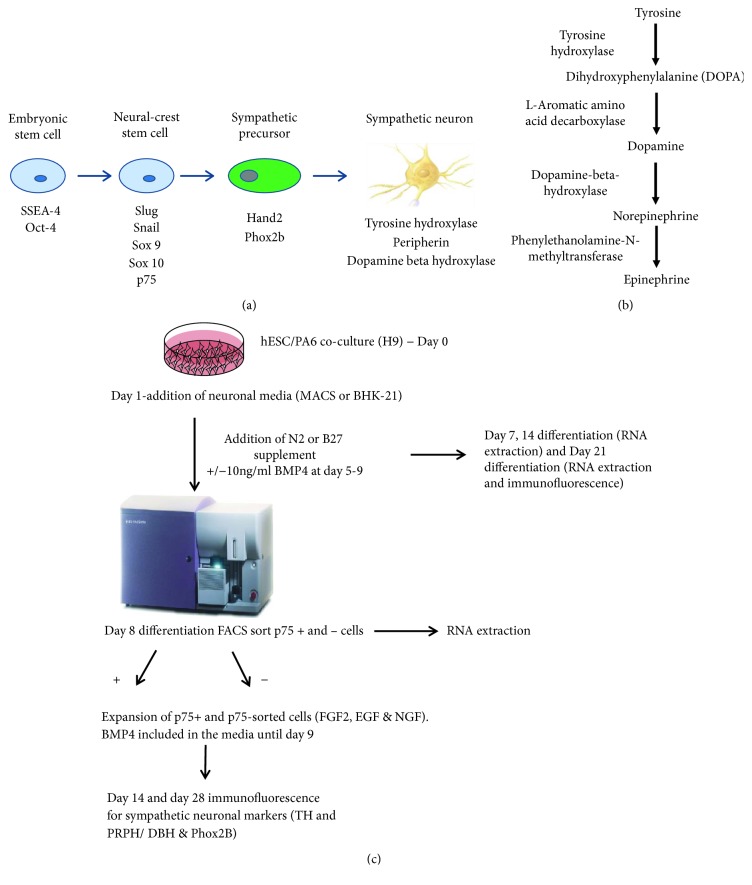
(a) Markers used to identify cell populations in this study. (b) The catecholamine biosynthesis pathway. (c) Flow chart detailing experimental outline of neural differentiation.

Sympathetic neurons originate from trunk neural crest cells that arrest their migration upon arrival at the dorsal aorta and begin to express the catecholaminergic and noradrenergic biosynthetic enzymes tyrosine hydroxylase (TH) and dopamine betahydroxylase (DBH), respectively (Figures 1(a) and 1(b)). Bone morphogenetic proteins (BMPs), multifunctional secreted proteins of the transforming growth factor *β* superfamily, are secreted in the dorsal aorta and the gut [3] and are important for noradrenergic autonomic specification from the neural crest [4, 5].

Neuroblastoma is an embryonal malignancy originating from neural crest cells which give rise to the sympathetic nervous system (SNS) [3]. It is the most common childhood solid tumour outside the central nervous system, and in contrast to many other paediatric malignancies, high-risk neuroblastoma is fatal in around 50% of patients despite intensive multimodal therapy [6]. *In vivo* and *in vitro* observations have shown that neuroblastic tumours appear to recapitulate the development of differentiating, predominantly noradrenergic, sympathetic neurons, and chromaffin cells of the adrenal medulla, suggesting that neuroblastoma arises from aberrant or blocked differentiation in normal SNS development (reviewed in [7]). By modelling the normal development of the neural crest and SNS, it may be possible to understand the pathogenesis of neuroblastoma and other abnormalities of the neural crest, e.g., neurocristopathies.

Human embryonic stem cells (hESCs) and induced pluripotent stem cells (IPSC) have the potential to provide an unlimited source of cells for both disease modelling and cell replacement therapy. The ability to differentiate hESC to neural crest-derived stem-like cells (NCDSC) and autonomic progenitors provides an important tool for modelling human neural crest development.

Kawasaki and colleagues were the first to demonstrate efficient induction of peripheral autonomic neuronal lineages from murine and primate hESC by coculture with PA6 cells, which possess stromal-derived inducing activity (SDIA) [8, 9]. Mizuseki et al. showed that early exposure of cocultured cells to BMP4 inhibited neural differentiation, whereas late exposure to high concentrations of BMP4 (days 5–9) induced differentiation to neural crest cells and autonomic progenitors [9]. Recently, other studies differentiating hESC have used BMP4 [10] or a feeder layer [11] to help induce SN differentiation.

The aim of this study was to develop an *in vitro* model using both BMP4 and a stromal feeder layer for efficient differentiation of hESC to noradrenergic sympathetic neurons (Figures 1(a) and 1(b)). We sought to determine the optimum conditions for the differentiation of hESC to SN by comparing different neural differentiation media, sorting methods for neural crest-like cells, and plating conditions for sorted cells.

Understanding normal SNS development in hESC models will enable us to learn more about the SNS as well as neural crest-derived malignancies such as neuroblastoma.

## 2. Materials and Methods

### 2.1. Cell Culture

H9 cells were obtained from the WiCell Bank (Wisconsin) following approval from the UK Medical Research Council (MRC) Stem Cell Steering Committee. Undifferentiated H9 hESCs [12] were cultured on either the human foreskin fibroblast cell line (NclFed(R)1A) [13], inactivated with 35Gy ionising radiation, or irradiated MEF-CF1 standard density cells (AMSBIO, UK). hESCs were cultured in stem cell media (20% KnockOut Serum Replacement (Invitrogen, USA), 0.1% nonessential amino acids (NEAA) (Invitrogen, USA), 0.1 mM *β*-mercaptoethanol (Invitrogen, USA), 2 mM Glutamax (Invitrogen, USA), and 8 ng/ml FGF2 (Invitrogen, USA) in KO-DMEM (Invitrogen, USA)). Cells were passaged weekly and replated on 6-well plates coated with irradiated feeder cells at a density of 6.5 × 10^3^ cells per well. The mouse stromal PA6 cell line was obtained from the Riken Cell Bank (Japan).

All cell lines were checked regularly and found to be free from contamination with Mycoplasma. Karyotypic analysis of H9 cells was also undertaken to confirm their identity using standard Giemsa banding techniques.

SKNAS (S-substrate adherent type, non-*MYCN* amplified) [14], IMR32 (N-neuronal type, *MYCN* amplified [15]), and SHSY5Y (N type, non-*MYCN* amplified) [16] human neuroblastoma cell lines were used as controls.

### 2.2. Differentiation to Neural Crest-Like Cells and Sympathetic Progenitors

Neural crest differentiation was induced by coculture of hESC with PA6 cells in neural differentiation media as outlined in Figure 1(c). Cells were detached from Fed1A feeders using 1 mg/ml collagenase IV and incubated for 10 minutes at 37°C to detach hESC colonies. 500–800 cells were transferred to 12- or 24-well plates, each well containing 1 or 0.5 × 10^4^ PA6 cells, respectively, and cultured for up to 28 days.

To optimise differentiation to p75+ve neural crest-like cells, two neural differentiation media were compared: (1) neural BHK media (90% BHK-21 medium/Glasgow modified Eagle medium (MEM) with 10% KO-SR, 1% L-Glutamax, 0.5% NEAA, 1% pyruvate, 1% penicillin/streptomycin, and 2 × N2 neuronal supplement) and (2) 90% MACS® neuronal media plus 2% MACS B27 supplement, 1% L-Glutamax, 0.5% NEAA, and 1% penicillin/streptomycin. Conditions required for optimal development of noradrenergic sympathetic neurons were established by comparing the addition or withdrawal of 10 ng/ml BMP4 and 10 ng/ml BMP2 and 4 with 10 ng/ml BMP4 alone, on days 5 to 9 of differentiation. N2 supplement (Life Technologies) and 10 ng/ml nerve growth factor (NGF) (R&D systems) were added to the media from day 4 of differentiation and 0.1 mM dibutyryl cyclic AMP (dbcAMP) was added on day 8 and withdrawn from the media after 10 days of differentiation. Cells were further differentiated for 3–4 weeks, and media were changed every two days. Differentiation experiments were carried out to *n* = 3.

### 2.3. p75 (CD271) Fluorescence-Activated Cell Sorting (FACS) of Differentiating Cells

Two methods of cell sorting for p75-positive cells were used to compare the yield of p75-positive cells obtained.

H9 cells were harvested on day 8 of PA6 coculture, which was found to yield the optimal number of p75-positive cells (data not shown) using 1 mg/ml collagenase, incubated for 10 minutes at 37°C, followed by incubation in Accumax (Stemgent) for 10 minutes with gentle agitation to obtain a single cell suspension. Cells were washed once in FACS wash buffer (1 in 20 dilution of bovine serum albumin (Miltenyi Biotec, UK) in MACs rinsing solution (Miltenyi Biotec) and centrifuged at 150 *g* for 4 minutes. 10% Fc block in FACS wash buffer was added to the cell suspension and incubated for 10 minutes at room temperature. Anti-p75 (CD 271) primary antibody directly conjugated with phycoerythrin (PE) (Miltenyi Biotec) was added to cells at 1 in 33 dilution. Multiple cell sorts were performed (*n* = 3) using a FACS Aria II Cell Sorter (BD Bioscience™) and a minimum of 5.5 × 10^4^ p75+ ve and p75– ve cells plated onto either PA6-coated 24 well plates or BD BioCoat™ poly-l-ornithine/laminin-coated 24 well plates (BD Biosciences). All sorted cells were cultured in MACS neuronal medium containing B27 supplement, 10 ng/ml NGF, 10 ng/ml fibroblast growth factor (FGF2), and 10 ng/ml epidermal growth factor (EGF).

### 2.4. Neural Crest Stem Cell (NCSC) Microbead Sorting

Differentiating H9 cells were harvested using Trypsin/EDTA and dissociated to a cell suspension. Positive cell enrichment was performed using anti-p75- (CD271) coated NCSC MicroBeads, according to the manufacturer's instructions (Miltenyi Biotec). Sorted cells were cultured in either 24-well PA6-coated plates or BD BioCoat™ poly-l-ornithine/laminin-coated 24-well plates.

### 2.5. Live Cell Immunofluorescence of Undifferentiated H9 Cells

Undifferentiated H9 hESC colonies were immunostained with 1 : 100 dilutions of TRA-1-60-FITC conjugate (Millipore) and anti-SSEA-4/clone MC-813-70-PE conjugate (Millipore). HESC colonies were incubated with primary antibodies at 37°C for 2 hours followed by a 10-minute incubation with 0.5 *μ*g/ml Hoechst-hESC media solution and twice washed with hESC media to ensure all Hoechst dye was removed. The colonies were then imaged in hESC media under a Nikon eclipse TE2000U inverted microscope after which the media was replaced with fresh hESC media containing 10 *μ*M Rho-associated kinase (ROCK) inhibitor Stemolecule™ Y27632 [17] (Stemgent, MA, USA).

### 2.6. Immunofluorescence of Differentiating Cells

H9 cells were immunostained for stem cell and neuronal markers. Cells were washed in PBS and fixed in 4% paraformaldehyde for 10 minutes. After washing in PBS (3 × 5-minute washes), the samples were incubated in blocking solution containing 1% BSA and 10% goat serum. The following antibodies were used at the dilutions indicated: OCT4 1 : 400 (Abcam), NANOG (R&D systems) 1 : 200, neural cell adhesion molecule (NCAM) 1 : 200 (Millipore), peripherin (PRPH) 7C5 and C-19 1 : 200 (Santa Cruz Biotechnology), TH 1 : 450 (Millipore), DBH, 1 : 450 (Abcam), and paired like homeobox2B (PHOX2B) 1 : 450 (Santa Cruz Biotechnology). Cells were incubated with primary antibodies overnight at either 4°C or room temperature for 1.5 hours. Secondary antibodies coupled to Alexa Fluor 488 or 568 (Molecular Probes, USA) were used for detection and were used alone as controls for two-colour costaining as well as comparison with single markers alone. Cells were washed with 3 × 10-minute washes and nuclei stained using 4′,6-diamidino-2-phenylindole, dihydrochloride DAPI (Vectashield) diluted 1 : 10 in PBS in 24-well plates, and coverslips were stained with DAPI. 24-well plates and 4-well chamber slides (Millipore, UK) were viewed and photographed using a Nikon A1r confocal microscope. Percentages of positively immunostained cells were obtained by counting 100 cells each in 3 different areas of the slide and then scoring the number of positive cells. This scoring process was applied to all experimental replicates.

### 2.7. Time Lapse Photography

Live cell analysis and imaging of p75+ and p75− H9 cells were performed over 4 days using a Nikon Biostation Cell Tracker. Cells were imaged on both PA6-coated 24-well plates and poly-l-ornithine/laminin 24-well plates. The migration rates, including velocity and meandering index, were measured using the Volocity™ software programme (Perkin Elmer, UK).

### 2.8. RNA Analysis-RT-PCR

RNA was extracted using the RNeasy mini kit (Qiagen) and 0.5 *μ*g reverse transcribed using the iscript cDNA synthesis kit (BioRAD™). RNA was isolated from differentiated H9 cells on day 7, 14, and 21 of differentiation. Undifferentiated stem cells and SKNAS, SHSY5Y, and IMR-32 neuroblastoma cell lines were used as controls. In addition, normal human adrenal cortex, medulla, and dorsal root ganglion tissue from a 14-week gestation fetus were supplied by the Human Developmental Biology Resource (http://www.hdbr.org).

RT-PCR reactions were set up in a total volume of 20 *μ*l, containing 10% PCR buffer, 10% magnesium chloride (MgCl_2_), 10% dNTPs, 1 *μ*l of 10 *μ*M forward and reverse primers, and 1% Amplitaq Gold™ (Applied Biosystems). RT-PCR was performed for neural crest specifiers (*SNAIL*, and *SOX9*), SNS precursors, (*PHOX2b* and *TH*), noradrenergic sympathetic neurons (*DBH*), and other neuronal markers (*NCAM*, *PRPH*) on days 7, 14, and 21 of coculture of unsorted cells, as well as on day 8 for p75+ ve FACS sorting (see Supplementary Information 1 for primer sequences). Densitometry was performed using ImageJ software (NIH, USA). Total intensity was calculated from PCR bands and first normalised to *GAPDH*; mRNA expression for each target was then calculated as fold change relative to undifferentiated H9 stem cells.

### 2.9. Quantitative Reverse Transcriptase PCR (QRT-PCR)

Taqman® gene expression assay primers and probes were used to amplify *OCT4*, *TP53*, *DBH*, and *GAPDH*. RT-PCR was performed in a total reaction volume of 10 *μ*l containing 5 *μ*l of Taqman universal PCR master mix, 0.5 *μ*l of primers and probes mix (Applied Biosystems), 2.5 *μ*l cDNA, and 2 *μ*l H_2_O. Reactions were performed in triplicate then quantified using the ABI Prism 7900HT sequence detection system (Applied Biosystems) relative to *GAPDH*.

### 2.10. Statistics

A chi-squared test was used to compare the percentages of p75+ and p75− ve cells obtained using MACS® neuronal media and neural BHK media (*n* = 3).

Continuous velocity data for p75+ cells grown on PA6 cells v poly-l-ornithine/laminin plates was summarised using the mean and standard deviation for normally distributed data and medians with quartiles for skewed data. Log transformations were performed to reduce skewness, and two sample *t*-tests were used to compare normally distributed data. The statistical package STATA version 14.1 (StataCorp 2015, Stata Statistical Software: Release 14; College Station, TX: StataCorp LP) was used for statistical analyses.

## 3. Results

### 3.1. Confirmation of Phenotype and Karyotype of H9-Undifferentiated hESC

**Figure 2:**
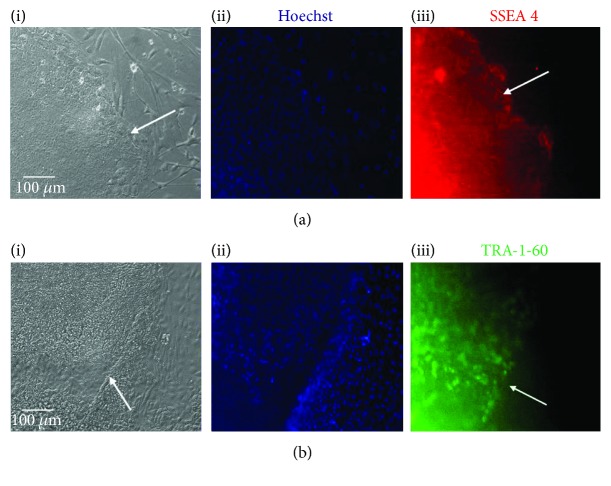
H9 hESC live cell staining for pluripotency markers SSEA4 and TRA-1-60. (a-i, b-i) Phase contrast microscopy. (a-ii, b-ii) Hoechst staining showing efflux of Hoechst from stem cells but not feeder layer. (a-iii) SSEA-4 (red) and (b-iii) TRA-1-60 (green) showing specific staining for human ES cell colonies compared with control feeder cells. White arrows highlight the stem cell colony borders.

Live cell staining using antibodies specific to the pluripotency markers SSEA4 and TRA-1-60 showed intense staining of undifferentiated H9 ES cell colonies (Figures 2(a-iii) and 2(b-iii)). Hoechst staining of H9 cells was less intense than in the Fed1A feeder cell layer, indicative of a “Hoechst dim” phenotype due to efflux of Hoechst dye by stem cells, in contrast to brighter staining observed on the feeder layer (Figures 2(a-ii) and 2(b-ii)). Karyotyping of H9 cells confirmed a normal female karyotype (Figure S1).

### 3.2. Sympathetic Neuronal Differentiation of H9 Cells Detected by Immunofluorescence and RT-PCR

**Figure 3:**
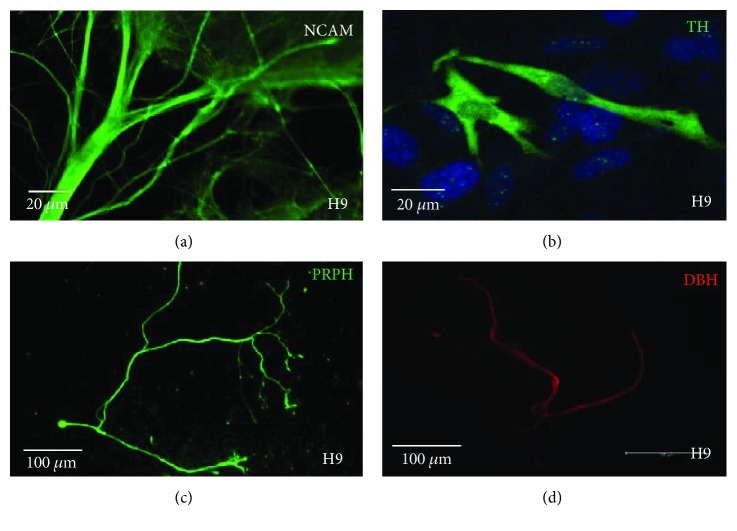
Immunofluorescence of unsorted H9 hESCs on day 21 of neuronal differentiation demonstrating expression of (a) neural cell adhesion molecule (NCAM), (b) tyrosine hydroxylase (TH), (c) peripherin (PRPH), and (d) dopamine betahydroxylase (DBH).

Following 7 days of PA6 coculture, morphological changes towards a neuronal phenotype were observed in H9 cells using phase contrast microscopy. Immunofluorescence of H9 cells on day 21 of differentiation for neuronal markers showed >90% of cocultured cells immunostained positive for NCAM (Figure 3(a)) with PRPH positivity in 20% (Figure 3(c)). TH-positive cells were observed in around 10% of the cell population (Figure 3(b)). DBH was detected in approximately 5% of differentiated cells (Figure 3(d)). This cell population included around 10% PA6 cells, and thus, the percentage of cells differentiating towards a sympathetic neuronal lineage is likely to be slightly higher than this.

**Figure 4:**
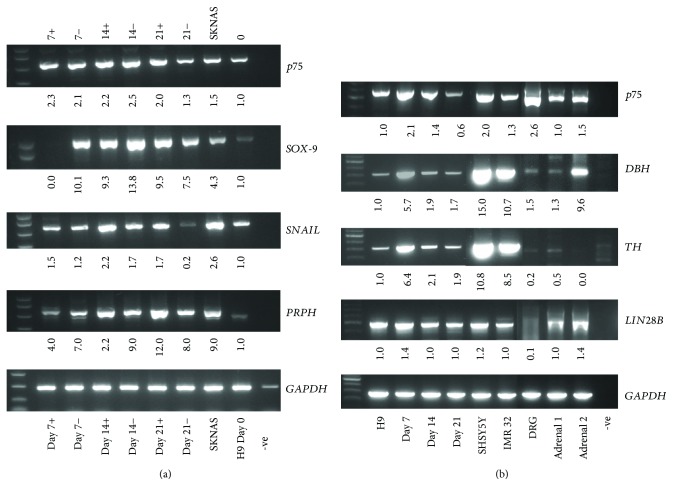
Neuronal differentiation in H9 cells. (a) Semiquantitative RT-PCR showing mRNA expression of neural crest specifiers *p75*, *SOX-9*, and *SNAIL* and the neuronal marker *PRPH* on days 0, 7, 14, and 21 of differentiation in the presence (+) or absence (−) of 10 ng/ml BMP4 on days 5–9 of differentiation with the SKNAS neuroblastoma cell line as a + control. (b) RT-PCR expression of *p75*, *DBH*, *TH*, and *LIN28B* expression in undifferentiated H9 cells and days 7, 14, and 21 of differentiation compared with control SHSY5Y and IMR-32 neuroblastoma cell lines, normal fetal adrenal gland (1 and 2) and fetal dorsal root ganglion. −ve = negative control.

mRNA expression by RT-PCR of early neural crest specifier genes including *SNAIL* showed a twofold increase between day 0 and day 14, and *SOX-9* expression increased 14-fold by day 14 (Figure 4(a)). The addition of BMP4 (10 ng/ml) on days 5–9 of coculture led to a 12-fold increase in *PRPH* expression from day 7 to day 21 of differentiation compared to 8-fold without BMP4 (Figure 4(a)). *p75* expression was highest between day 7 and day 14 of differentiation and increased upon addition of BMP4 (Figures 4(a) and 4(b)). Low basal expression of *p75*, *DBH*, and *TH* was detected, possibly due to spontaneous differentiation into neuronal cells as has previously been reported for H9 hESC [18]. High expression of *p75* was observed in the neural crest-derived control tissues, fetal adrenal gland comprising adrenal cortex and medulla and control dorsal root ganglion (sensory neurons). There was a 5-fold increase in *DBH* expression and a 6-fold increase in *TH* expression by day 7 of differentiation compared to control H9 cells. High expression of *TH* and *DBH* was observed in the positive controls (IMR32 and SHSY5Y cells). *DBH* expression on day 7 was comparable with one of the fetal adrenal glands, and as expected, the fetal dorsal root ganglion showed low expression of *DBH* (Figure 4(b)). The highest expression of the pluripotency gene *LIN28B* expression was observed in H9 cells on day 7 of differentiation (Figure 4(b)).

### 3.3. Quantitative RT-PCR of Differentiating H9 Cells

**Figure 5:**
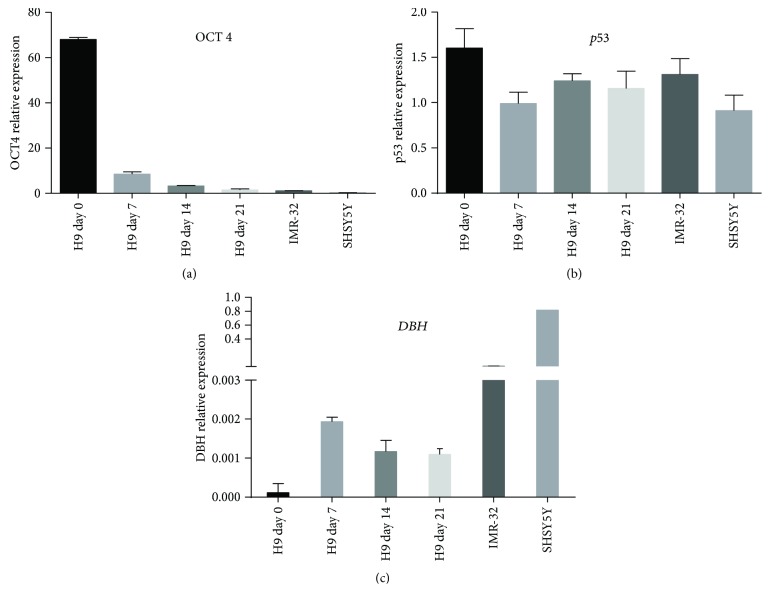
Quantitative RT-PCR of H9 cells. (a) High mRNA expression of *OCT4* in pluripotent H9 cells decreases throughout neuronal differentiation with low expression in SHSY5Y and IMR-32 neuroblastoma cell lines. (b) Sustained *TP53* expression throughout differentiation at levels similar to SHSY5Y and IMR-32 neuroblastoma cell lines. (c) Low *DBH* expression in undifferentiated H9 cells increases to a maximum on day 7 of differentiation but is less than SHSY5Y and IMR32 neuroblastoma cell lines (*n* = 3 in triplicate, values relative to *GAPDH*).

The highest expression of the pluripotency marker *OCT4* was observed in undifferentiated H9 cells, decreasing by day 7 through day 21 of differentiation (Figure 5(a)). SHSY5Y and IMR-32 neuroblastoma cell lines expressed low levels of *OCT4* compared with undifferentiated H9 cells (Figure 5(a)). *TP53* was expressed in pluripotent H9 cells and expression maintained throughout differentiation at levels comparable with the neuroblastoma cell lines (Figure 5(b)). *DBH* expression was very low in undifferentiated H9 cells and increased to a maximum on day 7 of differentiation before declining during the later stages of differentiation (Figure 5(c)), consistent with the data obtained by RT-PCR where the highest expression of *DBH* was also on day 7 of differentiation (Figure 4(b)). As expected, control neuroblastoma cell lines expressed very high levels of *DBH* (Figure 5(c)).

### 3.4. Enrichment of Sympathetic Progenitor-Like Cells by p75 Sorting

**Figure 6:**
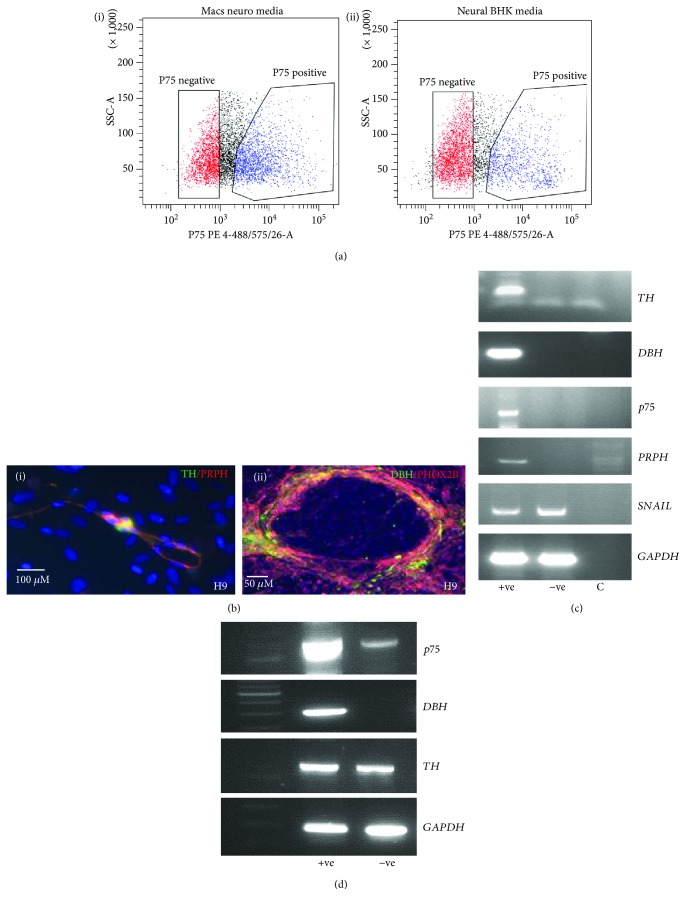
(a–c) Fluorescence-activated cell sorting (FACS) for p75 (neural crest stem cell marker). (a) FACS plots for H9 cells sorted on day 8 of differentiation. (a-i) 44.7% ± 6.1 p75+ viable cells in MACS® neuronal media. (a-ii) 32.5% ± 1.4 p75+ viable cells in neural BHK media. (b) Immunofluorescence of p75+ FACS-sorted H9 cells at different stages of differentiation. (b-i) PRPH and TH copositive H9 cells at 14 days post cell sort. (b-ii) PHOX2B and DBH copositive H9 cells at 14 days post cell sort. (c) mRNA expression by RT-PCR of FACS sorted H9 p75+ and p75− ve fractions showing high expression of *TH*, *DBH*, and *PERIPHERIN* in p75+ cells and undetectable expression in p75− cells and equivalent *SNAIL* expression in both populations. (d) RT-PCR of p75-enriched H9 cells isolated using anti-human p75 antibody-coated magnetic MicroBeads showing *TH* and *p75* expression in p75-depleted population. +ve = p75 positive fraction, −ve = p75 negative fraction, and c = negative control.

To enrich for NCDSC and sympathetic neuron-like cells, cells were sorted using FACS for the NCSC marker p75 (CD271) on day 8 of coculture (Figure 4(b)). To optimise neural crest stem cell differentiation further, two different neuronal media types were compared: (a) MACS® neuronal media + B27 and (b) neural BHK media + N2 supplement. On day 8 of differentiation, an increased proportion of p75+ ve cells were observed using MACS® neuronal media 45.0% ± 0.8 (95% confidence interval) compared with 32.7 ± 0.8 (95% confidence interval) using neural BHK media (*n* = 3, *p* < 0.0001, chi-squared test) (Figure 6(a)). Preliminary results undertaking FACS for both p75 and DBH on day 8 of differentiation showed that 5.5 and 5.7% of cells were p75 and DBH+, by FACS following culture in BHK and MACS media, respectively (data not shown).

Following p75 sorting, autonomic neuronal populations were enriched when cells were grown in preconditioned media from cultured hESC in the presence of N2 supplement, NGF, and dbcAMP (data not shown). On day 28 post p75 sorting, dual immunofluorescence identified sympathetic neurons by PRPH and TH copositivity cells in H9 cells (Figure 6(b-i)) in up to 20% cells. Noradrenergic sympathetic neurons identified by copositivity for both PHOX2B and DBH were present in 9.4% ± 5.5% H9 cells (Figure 6(b-ii)). mRNA expression by RT-PCR of p75+ ve H9 cells showed enrichment for *p75* and the sympathetic neuronal markers, *TH* and *DBH*, together with *PRPH* where expression was observed exclusively in p75+ ve cells (Figure 6(c)). This was in contrast to the early neural crest specifier *SNAIL* which was expressed in both p75+ ve and p75− ve cells. Following selection of p75+ ve cells using neural crest stem cell microbeads in differentiating H9 cells, *p75* mRNA expression was highly enriched in the p75+ ve cell fraction and *DBH* was expressed exclusively in p75+ ve cells whereas *TH* was expressed in both the p75-enriched and the p75-depleted cell populations (Figure 6(d)). This indicates possible contamination of p75+ ve cells in the p75-depleted population.

### 3.5. Live Cell Imaging (Biostation)

**Figure 7:**
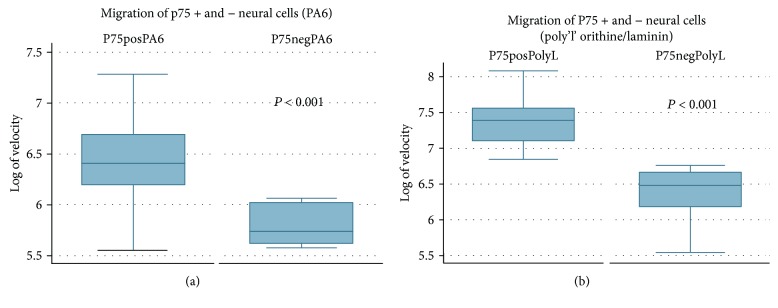
Migration of p75+ and p75– H9 cells sorted by FACS on (a) PA6 coated wells showing a higher mean logged velocity of p75+ cells compared with p75− cells (*p* < 0.001), and (b) poly ‘L'ornithine/Laminin coated plates again showing a higher mean logged velocity in p75+ cells (*p* < 0.001).

p75+ ve-sorted cells plated onto PA6-coated plates cells survived better compared to those on poly-l-ornithine/laminin-coated plates (data not shown). H9 cells appeared to track and follow PA6 cells adhering to them which in turn led to enhanced survival. Time-lapse microscopy revealed that p75+ ve cells showed increased migration compared with p75− ve cells. A *t*-test for independent groups with correction for unequal variances showed that the mean logged velocity for p75+ ve cells grown on PA6 cells was higher than that for p75− ve cells (*p* < 0.001). A similar analysis showed that the mean logged velocity for p75+ ve cells grown on poly-l-ornithine /laminin cells was higher than that for p75− ve cells (*p* < 0.001) (Figures 7(a) and 7(b); Supplementary online video 1).

## 4. Discussion

The aim of the current study was to optimise a model of normal human sympathetic neuronal development using hESC, which could be used to understand the normal development of the SNS and in the future the pathogenesis of neuroblastoma and other neural crest-derived malignancies.

In this study, we optimised derivation of neural crest-like cells and noradrenergic sympathetic neuron-like cells using a variety of methods. Initially, we tested conditions for neural crest-derived stem line cell differentiation by comparison of two media types: (1) MACS® neuronal media and (2) neural BHK media, where the former was found to be superior for the generation of p75+ ve cells. We also optimised BMP exposure and showed that BMP4 exposure alone was superior, in agreement with previous studies showing that although early exposure to BMP4 can promote dorsal neural differentiation, when applied at later stages, BMP4 enhances the production of NCSC and autonomic neurons in primate and murine cells [9, 19]. BMP signalling is essential for the initiation of differentiation of neural crest cells into sympathetic neurons in the developing embryo [20, 21]. To our knowledge, there is only one other study differentiating hESC to autonomic neurons which has employed the use of BMP4 during differentiation [10], but this study did not use SDIA which is likely to be at least partly responsible for the higher yields of sympathetic neurons we observed (Table 1).

p75 is the low affinity NGF receptor and a well-characterised marker for neural crest-derived stem-like cells [22]. Using a murine in vivo model, NGF was shown to bind the high-affinity NGF receptor (TRKA) which regulates the expression of both TH and DBH in developing and maturing sympathetic neurons [23]. p75 cell sorting has been used previously to purify neural crest stem cells from hESCs [24, 25], and our work has extended this field by showing that day 8 of differentiation induced by SDIA is the optimal time for p75 cell sorting.

The presence of TH and PRPH costaining or DBH and PHOX2B was used to identify catecholaminergic and noradrenergic sympathetic neurons, respectively. TH is the rate-limiting enzyme in the biosynthesis of dopamine and noradrenaline and is a useful marker for catecholaminergic neurons (Figure 1(b)) [26]. PRPH is expressed in neurons of the developing peripheral nervous system [27].

PHOX2B regulates the expression of PHOX2A and heart and neural crest-derived expressed protein 2 (HAND2). Hand2 is induced by BMPs and is first observed after the onset of Phox2B and Asc-1 expression. Overexpression of Hand2 has been shown to induce the generation of catecholaminergic neurons from neural precursor cells both in vitro and in vivo [4, 5]. Furthermore, germline mutations in *PHOX2B* have been identified in hereditary neuroblastoma [28, 29]. DBH, a specific marker of noradrenergic sympathetic neurons, is expressed in some neuroblastoma cell lines [30]; it catalyses the conversion of dopamine to noradrenaline in the catecholamine synthesis pathway leading to noradrenergic neurons [31]. In the current study, noradrenergic sympathetic neurons were identified by *DBH* mRNA expression and immunostaining for DBH alone and DBH/PHOX2B copositivity.

Time-lapse analysis of p75+ ve and p75− ve cells showed that p75+ ve cells had increased migration compared with p75− ve cells, consistent with migratory properties of neural crest cells [1]. These results mirror findings observed in vivo using live cell imaging during embryogenesis [32].

QRT-PCR gene expression analysis showed high expression of the pluripotency markers *OCT4* and *LIN28B* in pluripotent H9 cells decreasing with the onset of neural differentiation as expected [35].

*TP53* expression was observed throughout differentiation at levels similar to those in neuroblastoma cell lines consistent with evidence that p53 regulates the proliferation and differentiation of neural progenitor cells independently of its role in the induction of apoptosis. *In vivo* studies using transgenic mouse models have demonstrated a fundamental role for p53 during neural stem cell self-renewal and differentiation [36, 37].

Previous studies have used murine neural crest systems to investigate neuroblastoma development by *MYCN* transformation of primary neural crest cells derived from day 9.5 mouse embryos [38]. Further studies also reported that *MYCN* and common *ALK* mutations exert a role in neuroblastoma tumour initiation using neural crest progenitor cell lines MONC-1 and JoMa1 [39, 40]. Very recently, human NCSC derived from hESC have been transformed by *MYCN* to form neuroblastoma in vivo [11]. The use of human stem cell models of sympathoadrenal development such as ours will develop this field further.

In conclusion, our study describes advancement in the generation of noradrenergic sympathetic neuron-like cells from hESC to improve our understanding of the normal development of the human SNS and abnormalities thereof including neural crest-derived malignancies such as neuroblastoma. This model could later be perturbed by oncogenic transformation of these cells with genes known to be important in the development of neuroblastoma including MYCN and/or ALK as has been reported for NCDSC, to better understand events leading to the development of neuroblastoma, its cell of origin, and new potential treatment targets.

## Figures and Tables

**Table 1:** Comparison of the current study with previously published studies reporting the differentiation of pluripotent stem cells to sympathetic neurons.

Reference	Methods	Cell type	Markers used to identify SNS progenitors	Yield
Mizuseki et al. [9]	PA6 stromal cells/BMP4	mESCs, primate ESCs	TH+/PRPH+/Phox2b	Not quantified—very low
Pomp et al. [33]	PA6 coculture BHK-21 medium/Glasgow MEM + N2 supplement	HES1, HUES1, HUES7	TH+/PRPH+	<1%
Lee et al. [25]	MS5 stromal cell line 28 days, FGF2/EGF exposure	H9	TH+/PRPH+	1-2%
Jiang et al. [24]	PA6 stromal cell line—7 days BHK-21 medium/Glasgow modified Eagle's medium + N2 supplement	H1, H9	TH+/PRPH+	18%
Acevedo et al. [34]	Embryoid bodies (EBs)—onto collagen plates	H9	Dopa decarboxylase (DDC), TH+, Mash 1	Not determined
Huang et al. [10]	Exposure to retinoic acid, BMP2, BMP4, BMP7	H1, WTC iPSC	TH+/DBH+	Not determined
This study	PA6 stromal cells, MACS® neuronal media + BMP4	H9	TH+/PRPH+, DBH+/PHOX2B+	20% and 9.4% ± 5.5%, respectively

## Data Availability

The data used to support the findings of this study are available from the corresponding author upon request.

## Abbreviations

BMP: Bone morphogenetic protein
hESC: Human embryonic stem cells
DBH: Dopamine betahydroxylase
NCAM: Neural cell adhesion molecule
SDIA: Stromal-derived inducing activity
PRPH: Peripherin
SNS: Sympathetic nervous system
TH: Tyrosine hydroxylase.

## Supplementary Materials

Supplementary 1 Figure S1: G-banded karyotype analysis of H9 hESC showing a normal female karyotype.

Supplementary 2 Video 1: p75+ FACS-sorted H9 cells showing motile p75+ cells tracking the adherent PA6 feeder cells over a 12-hour time period between days 8 and 12 of differentiation.

Supplementary 3 Supplementary Information 1: primer sequences (forward and reverse) including product length and annealing temperatures are detailed below 5′ to 3′.
