# Supplementary figures and images for: Differentiation of Human Embryonic Stem Cells to Sympathetic Neurons: A Potential Model for Understanding Neuroblastoma Pathogenesis
Source: Stem Cells Int. 2018 Nov 1;2018:4391641. doi: 10.1155/2018/4391641 (PMC6236576; doi:10.1155/2018/4391641)

Supplementary Figure 1

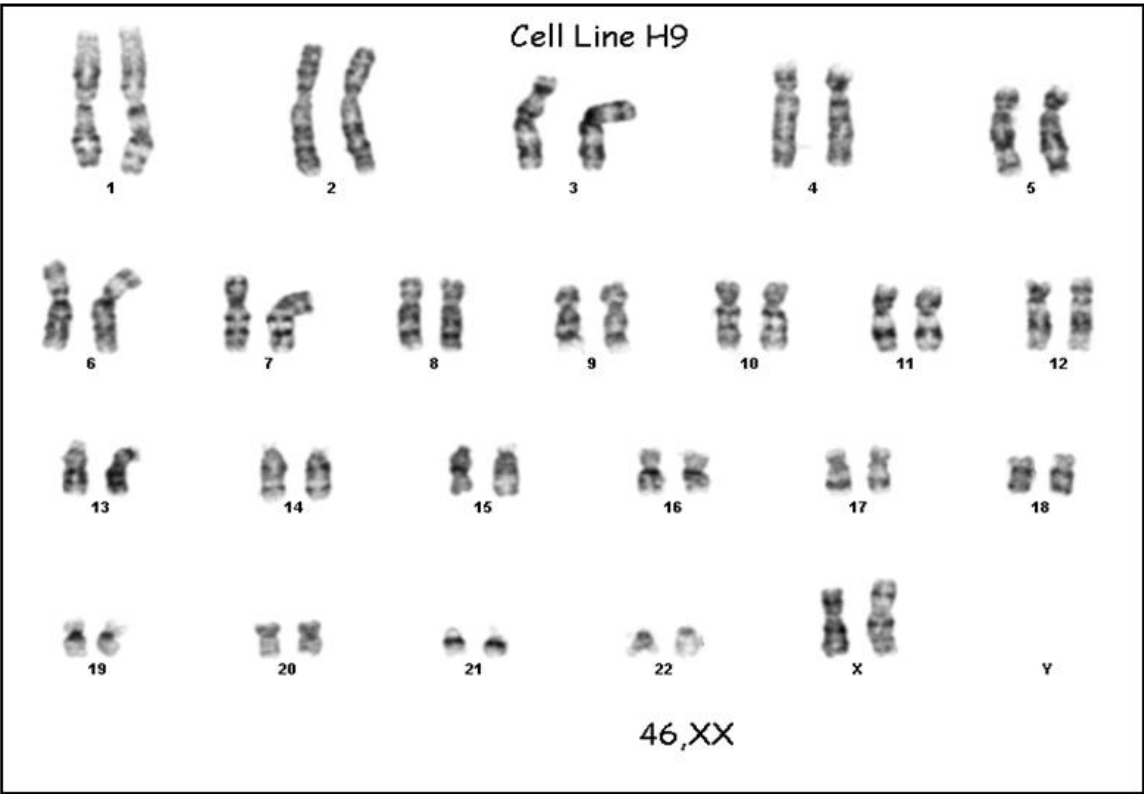

Supplement: Supplementary 1 — Figure S1: G-banded karyotype analysis of H9 hESC showing a normal female karyotype. [file 4391641.f1.pdf]
