# Supplementary material for: Differentiation of Human Embryonic Stem Cells to Sympathetic Neurons: A Potential Model for Understanding Neuroblastoma Pathogenesis
Source: Stem Cells Int. 2018 Nov 1;2018:4391641. doi: 10.1155/2018/4391641 (PMC6236576; doi:10.1155/2018/4391641)
Supplement: Supplementary 3 — Supplementary Information 1: primer sequences (forward and reverse) including product length and annealing temperatures are detailed below 5′ to 3′. [file 4391641.f3.docx]

**Supplementary information 1**

*SOX9* (Forward; TTGTTTACAATAAATATACATTGC, Reverse; GCAATGTATATTTATTGTAAACAA, 294 bp) Annealing temp - 58^o^C

*SNAI1* (Forward; CTCCTCTACTTCAGCCTCTT, Reverse; CTTCATCAAAGTCCTGTGGG, 611 bp) Annealing temp – 55^o^C

*p75* (Forward; CCCCCTTCTCCCACACTGCTA, Reverse; GAACCCCAAACCTGACTCCAT 591 bp) Annealing temp - 55^o^C

*GADPH* (Forward; CTTTTAACTCTGGTAAAGTGG, Reverse; TTTTGGCTCCCCCCTGCAAAT, 287 bp) Annealing temp - 55^o^C

*DBH* (Forward; GACTCAACTACTGCCGGCACGT-3, Reverse; CTGGGTGCACTTGTCTGTGCAGT-3) Annealing temp - 60^o^C

*PRPH* (Forward; TTGAGTTCCTCAAGAAGCTGCACG, Reverse; CACCTCAGGCACAGTCGTCGTCTTTAT) Annealing temp – 56^o^C

*TH* (Forward; ACTGGTTCACGGTGGAGTTC Reverse; AGCTCCTGAGCTTGTCCTTG ) Annealing temp - 56^o^C

*LIN28B* (Forward; TCTCACGAGTTTGGAGCTGAG, Reverse; AATGGCACTTCTTTGGCTGAG) Annealing temperature 56^o^C
